# COVID-19 and the potential of Janus family kinase (JAK) pathway inhibition: A novel treatment strategy

**DOI:** 10.3389/fmed.2022.961027

**Published:** 2022-08-30

**Authors:** Mansoor Khaledi, Fatemeh Sameni, Sheida Yahyazade, Maedeh Radandish, Parviz Owlia, Nader Bagheri, Hamed Afkhami, Mohamad Mahjoor, Zahra Esmaelpour, Maryam Kohansal, Farzad Aghaei

**Affiliations:** ^1^Department of Microbiology, Faculty of Medicine, Shahed University, Tehran, Iran; ^2^Department of Immunology, School of Medicine, Isfahan University of Medical Sciences, Isfahan, Iran; ^3^Molecular Microbiology Research Center, Faculty of Medicine, Shahed University, Tehran, Iran; ^4^Cellular and Molecular Research Center, Basic Health Sciences Institute, Shahrekord University of Medical Sciences, Shahrekord, Iran; ^5^Shahed University, Tehran, Iran; ^6^Department of Immunology, Faculty of Medicine, Iran University of Medical Sciences, Tehran, Iran; ^7^Reference Laboratory for Bovine Tuberculosis, Razi Vaccine and Serum Research Institute, Karaj, Iran; ^8^Department of Medical Biotechnology, Fasa University of Medical Sciences, Fasa, Iran

**Keywords:** COVID-19, JAK inhibitors, baricitinib, pacritinib, ruxolitinib, tofacitinib

## Abstract

Recent evidence proposed that the severity of the coronavirus disease 2019 (COVID-19) in patients is a consequence of cytokine storm, characterized by increased IL-1β, IL-6, IL-18, TNF-α, and IFN-γ. Hence, managing the cytokine storm by drugs has been suggested for the treatment of patients with severe COVID-19. Several of the proinflammatory cytokines involved in the pathogenesis of COVID-19 infection recruit a distinct intracellular signaling pathway mediated by JAKs. Consequently, JAK inhibitors, including baricitinib, pacritinib, ruxolitinib, and tofacitinib, may represent an effective therapeutic strategy for controlling the JAK to treat COVID-19. This study indicates the mechanism of cytokine storm and JAK/STAT pathway in COVID-19 as well as the medications used for JAK/STAT inhibitors.

## Introduction

Scientists have been familiar with the coronavirus for over half a century. The word “coronavirus,” which comes from the Latin corona (“crown”), was first used by Almeida to describe these viruses because their glycoprotein spikes resembled solar coronas (1, 2). In 1968, 229E and OC43 were found to be the first coronaviruses to infect people. However, it caused very minor infections until the severe acute respiratory syndrome (SARS-CoV-1) outbreak in 2003 and the Middle East respiratory syndrome (MERS) in 2012. Recently, the Chinese health authorities announced that there was widespread pneumonia of unknown cause in Wuhan, and the novel coronavirus genome was released and made available to the scientific community (3). The new coronavirus, tentatively called 2019-nCoV, is now called SARS-CoV-2 according to the International Committee on Classification of Viruses Research Group (4). Since, the emergence of SARS-CoV-2, over 298 million people have been infected and more than 5,469,000 deaths have occurred (5). The spike (S) glycoproteins of coronaviruses are the main targeting agents of antibodies and promote cell entry (6). cSARS-CoV-2 is spread through respiratory droplets, mainly during face-to-face contact. The mean time between exposure and onset of symptoms is 5 days, with 97.5% of them developing symptoms in 11.5 days. Fever, coughing, and shortness of breath are the most prevalent symptoms (7, 8). Asymptomatic carriers and severe clinical symptoms, which include septicemia and severe respiratory distress, are among the manifestations of COVID-19 (9). Severe symptoms requiring intensive care were experienced by about 5% of patients with COVID-19 and 20% of inpatients (10). The latest studies have shown that not only the extreme pulmonary headaches of SARS-CoV-2 but also gastrointestinal signs and symptoms and liver dysfunction were commonly detected in patients (11). Various immune sensors such as TLR7, RIG-I/MDA, and cGAS / STING contribute to the recognition of SARS-CoV-2. These sensors provoke an early response to interferon (IFN)-I, which is essential for infection control (12, 13). Neutralizing antibodies that bind to the angiotensin-converting enzyme 2 (ACE2) receptor spike (S) protein binding site may be the basis of defense, but the affinity of SARS-2 S protein's for ACE2 is much higher than that of (SARS-CoV-1), and its highly pathogenic challenges are shown in other studies (12, 14). The immune system of older people is slow, unprompted, and less effective, increasing their vulnerability to potential infections. High-risk groups have been shown to switch from congenital to adaptive inadequately, resulting in much lower antibody levels in these patients (12).

## Cytokine storm in COVID-19

Through immune cell recruitment and cytokine production, the immune system aids in the eradication of viral infections such as influenza and coronaviruses. Immune response coordination is usually the first line of defense for the body when it comes to fighting viral infections. If the abnormal or exaggerated immune response of the host is not regulated, it might lead to severe disease (15, 16). Based on multiple studies, the most critical manifestations of COVID-19 are caused by uncontrolled immune responses and hyperinflammation (17). Feng et al. presented that SARS-CoV-2 infection triggers a hyperinflammation condition, knowns as cytokine storm, that results in acute respiratory distress syndrome (ARDS), thromboembolic diseases, encephalitis, myocardial infarction, acute kidney injury, adult vasculitis, Kawasaki-like syndrome in children, and even death (18).

The cytokine storm is an uncontrolled systemic host inflammatory reaction to many stressors such as infection, malignancy, rheumatoid disorders, and drugs (15). This situation is known as the hyperactivation of immune cells and extreme production of massive inflammatory cytokines, chemokines, and other chemical mediators (19). The term cytokine storm was first stated in the context of graft vs. host disease (GVHD) in 1993 (20). It also has been reported in several viral infections, in addition to COVID-19, like influenza H5N1, influenza H1N1, and two other coronavirus family members, SARS and MERS infections (21–24).

**Figure 1:**
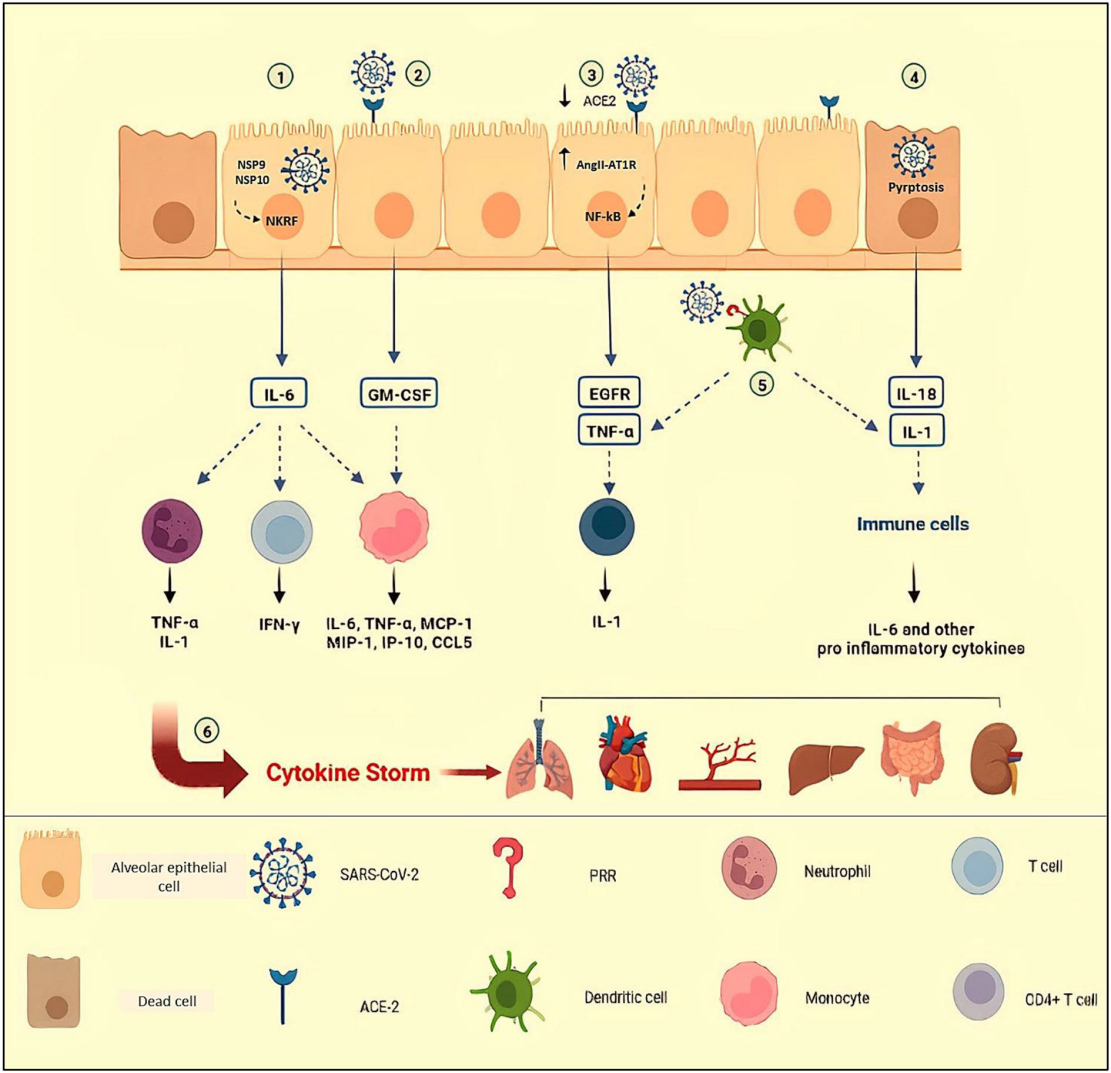
Some mechanisms of cytokine storm in COVID-19. (1) NSP9 and NSP 10 of SARS-CoV-2 target NKRF to promote IL-6 production. By engagement of receptor with IL-6, IFN-γ and GM-CSF are secreted by activated T cells; MCP-1, MIP-1, MIP-1, IP-1O, and CCL-5 are secreted by activated monocytes; and neutrophils release pro-inflammatory cytokines such as TNF-α and IL-1. (2) In response to SARS-CoV-2 in epithelial cells, secreted GM-CSF activates CD14+ CD16+ monocytes to produce pro-inflammatory cytokines such as IL-6 and TNF-α. (3) Attachment of SARS-CoV-2 to ACE-2, leads to a reduction in ACE-2 expression and accumulation of AngII in the host cells. The AngII and its receptor (AT1R) complex activate NF-κB, leading to the production of TNF-α and EGFR. (4) In COVID-19, increased levels of IL-1β and IL-18 indicate inflammasome activation and pyroptosis of host infected cells, triggering inflammation by recruiting immune cells and pro-inflammatory cytokine production. (5) Dendritic cells that are triggered by PRRs release TNF-α and IL-1, causing acute inflammatory responses. TNF/TNFR interactions between effector CD4+ T cells and myeloid cells have been suggested to promote IL-1β production. (6) Uncontrolled immune responses and hyperinflammation cause excessive production of pro-inflammatory cytokines, known as cytokine storms which are related to multi-organ failure and even death in COVID-19. NSP, Non-Structural Protein; NKRF, NF-κB repressor; ACE-2, Angiotensin Converting Enzyme-2; AngII, Angiotensin II; AT1R, Angiotensin II Receptor type 1; PRR, Pattern Recognition Receptors Created in Biorender.com.

Acute respiratory distress syndrome is a more notable consequence in severe episodes of cytokine storm in COVID-19, involving roughly 20–40% of hospitalized patients (25, 26). Hypercytokinemia and infiltration of activated monocytes, neutrophils, B cells, and T cells to the lungs result in diffuse alveoli–capillary membrane damage, increase lung permeability, pulmonary edema, hyaline membrane formation, micro thrombosis, fibrin exudates, and fibrotic healings resulting in the end to respiratory insufficiency (27–30). Based on the fact that the presence of SARS-CoV-2 particles in bronchial and alveolar type II epithelial cells have been revealed by electron microscopy, severe lung injury in COVID-19 may be the result of both direct viral infection and cytokine storm (31, 32). Some mechanisms of cytokine storm in COVID-19 have been summarized in Figure 1.

Following the entry of SARS-CoV-2 into the respiratory epithelial cells, the virus stimulates an immune response with inflammatory cytokine production accompanied by a weak IFN response (33, 34). In the early phases of viral infection, the synthesis of IFN-I or IFN-α/β is the main molecule that performs an antiviral effect (35). Recently, McGonagall et al. and Ye Q et al. presented an attractive concept of the cytokine storm in COVID-19. In these articles, the cytokine storm is known to be the result of immune system failure in removing the virus. In this case, the cytokine storm is divided into two stages: the first stage is an interim immune-deficient condition and the next is a hyperactive immune situation to compensate for the failure in reduction of viral load, which manifests as a cytokine storm (36, 37). The study of Hadjadj et al. is another confirmation that a failure in initial type-I and III IFN responses to SARS-CoV-2 leads to an excessive late immune response and a severe form of COVID-19 (38). On the other hand, IFN levels rose in COVID-19 in tandem with the viral load. The postponed peak, along with a drop in lymphocyte numbers, enhanced neutrophil infiltration into the lungs, worsening the patient's condition (39, 40). Also, similar to SARS-CoV-2, delayed release of IFNs in the early stages of SARS-CoV and MERS-CoV infection hampers the immune antiviral response. Following that, the rapid production of cytokines and chemokines results in an excessive infiltration of inflammatory cells into the lungs and, as a result, lung injury (41–43).

Analysis of cytokine levels in the serum of patients with COVID-19 in a study in China showed that the levels of IL-6, IL-1β, IL-7, IL-8, IL-9, IL-10, IL-2, FGF, G-CSF, GM-CSF, IFN-γ, IP-10, MCP-1, MIP-1α, MIP-1β, RANTES, PDGF, TNF-a, and VEGF are elevated compared to healthy controls. In addition, patients hospitalized in the ICU also exhibited increased cytokine levels of IL-2, IL-7, IL-10, G-CSF, IP10, MCP-1, MIF-1α, and TNF-α than those not requiring ICU treatment (39). Based on wide data, high levels of expression of IL-5, IL-1β, IL-4, IL-10, TNF-α, IFN- γ, IP-10, and MCP-1 have been detected in the serum of patients, and the levels of IL-2R and IL-6 are positively correlated with disease severity (39, 44, 45). The cytokine storm markers observed are not unique to SARS-CoV-2; comparable findings were made in SARS-CoV-1 and MERS-CoV cohorts (46, 47).

Among other cytokines, IL-6 has been at the center of the COVID-19 pandemic (48–50). Non-structural protein (NSP) 9 and NSP10 of SARS-CoV-2 target NKRF (NF-κB repressor) to promote IL-6/IL-8 production, according to a study of peripheral blood mononuclear cells (51). IL-6 levels were a significant predictor of illness severity early in the COVID-19 pandemic (50, 52, 53). Many documents revealed that those who died because of COVID-19 had higher serum levels of interleukin IL-6 compared to survivors (53, 54).

This cytokine is produced by stromal cells, B cells, T cells, macrophages, monocytes, dendritic cells, mast cells, and non-immune cells, such as fibroblasts, endothelial cells, keratinocytes, and tumor cells (55). IL-1β and TNF-α are two pro-inflammatory cytokines involved upstream of the IL-6 signaling pathway through the activation of NF-κB and, subsequently, the virus-induced cytokine storm (56, 57). In addition to activating inflammatory cells, NF-κB stimulation causes the production of IL-6, other pro-inflammatory cytokines, and chemokines (56, 58). IL-6 binds to either membrane-bound IL-6 receptor (mIL-6R) or soluble IL-6 receptor (SIL-6R), forming a complex that acts on gp130 (cytoplasmic signaling molecules), and triggers the JAK/STAT3 signaling pathway (59). STAT3 is required for NF-κB activation to be boosted. As a result, infection with SARS-CoV-2 activates both NF-κB and STAT3 (60, 61). In this way, mbIL-6R is expressed on naive T cells, monocytes/macrophages, and neutrophils. Following the engagement of receptor with IL-6 and subsequent JAK/SATAT3 signaling, IFN-γ and GM-CSF are secreted by activated T cells; MCP-1, MIP-1, MIP-1, IP-10, and CCL-5 are secreted by activated monocytes/macrophages; and neutrophils release pro-inflammatory cytokines such as TNF-α and IL-1β (62, 63).

In non-immune cells, the IL-6 amplifier (IL-6 Amp) is a hyper NF-κB activation mechanism caused by the simultaneous activation of NF-κB and STAT3. The IL-6 Amp stimulates the production of many proinflammatory cytokines, including IL-6, by hyperactivating NF-κB via STAT3. As a result, IL-6 Amp might be related to the cytokine storms in COVID-19 (61, 64).

In the case of COVID-19, IL-6 is one of the causes of lymphocytopenia *via* inhibiting lymphopoiesis through lymphocyte trafficking and the direct effect on progenitor cells (65, 66). The precursors for both lymphoid and myeloid fate express IL-6R. Followed by IL-6 binding, the JAK/STAT3 signaling leads to the expression of IL-6 in these cells, which suppresses lymphopoiesis (67). Chen et al. showed that SARS-CoV-2 selectively induced macrophages to produce IL-6 to directly promote lymphocyte death (68).

As a bridge between innate and adaptive immunity, IL-6 promotes TH17 and CD8+ T cells differentiation and activation, which themselves increase inflammation. In contrast, this cytokine inhibits the differentiation of regulatory CD4+ CD25+ FOXP3+ T cells (69–71).

Generally, regarding clinical symptoms, IL-6 induces cardiomyopathy, vascular leakage, activation of complement, and diffuse intravascular coagulation (72, 73).

Besides IL-6, IL-1 and TNF-α could be a predictor of cytokine storm and COVID-19 progression (74). Dendritic cells and macrophages that have been triggered by PRRs release these two cytokines, which causes acute inflammatory responses (75). TNF/TNFR interactions between effector CD4+ T cells and myeloid cells have been suggested to promote IL-1β production (76). Furthermore, in COVID-19, increased levels of IL-1β may indicate inflammasome activation and pyroptosis of host infected cells (77, 78). Microglia and IL-1 stimulation contribute to higher reactive oxygen species (ROS) and cytokine production, phagocytosis, and apoptosis inside the central nervous system (CNS) in the context of communication seen between the immune system cytokine network and the CNS cytokine network (79). Neuroinflammation, tissue damage, increased oxidative stress, and synaptic pruning dysfunction have all been reported as a result of this scenario. Inflammatory cytokines enter the circulation as well, potentially exacerbating the cytokine storm (80).

As SARS-CoV-2 enters the cells through ACE2, ACE2 expression in the cells is reduced and the serum levels of AngII increase. The AngII and its receptor (AT1R) complex activate NF-κB along with ADAM17, leading to the production of TNF-α, epidermal growth factor receptor (EGFR), and NF-κB stimulators, which is another way of TNF-α generation (81–83).

Increased expression of hyaluronic acid synthetase II in EpCAM+ lung alveolar epithelium, CD31+ lung alveolar endothelium, and fibroblasts in response to IL-1β and TNF-α leads to the accumulation of hyaluronic acid in the extracellular matrix and subsequent increase of fluid retention in airway spaces. This phenomenon creates the ground-glass appearance in the CT chest (84, 85).

Pneumonia is linked to blood levels of IL-1β, IL-7, IL-8, IL-9, and IL1-5 in COVID-19. These cytokines are produced from the injured tissue, and the same pattern was found in ICU and non-ICU patients, suggesting that they play a role in a cytokine storm. Furthermore, IL-2, IL-7, and IL-10 levels were shown to be higher in ICU and non-ICU patients (39, 86). To limit the cytokine storm, IL-10 levels are raised in the second week after symptoms start, but it is too late (87). In ICU patients, the levels of IL-4, a TH2 cytokine, and an anti-inflammatory suppressor are also raised (39).

GM-CSF is another cytokine involved in cytokine storm and is produced by macrophages, T-cells, fibroblasts, endothelial cells, epithelial cells, and tumor cells (88). In COVID-19, GM-CSF activates CD14+ CD16+ monocytes to produce pro-inflammatory cytokines such as IL-6 and TNF-α, further worsening the cytokine storm (89).

Data regarding the importance of chemokine dysregulation in COVID-19 has increasingly been reported. Transcriptome analysis of BALF samples indicated that the levels of pro-inflammatory chemokines such as CXCL1, CXCL2, CXCL6, CXCL8 (IL-8), CXCL10 (IP-10), CCL2 (MCP-1), CCL3 (MIP-1A), and CCL4 (MIP-1B) are elevated in patients with COVID-19, which correlated with disease severity. Enhanced expression of chemokine receptors such as CCR2 (CCL2 receptor) and CCR5 (CCL3 receptor) was also found, indicating that inflammatory signaling had been activated (90). High levels of macrophage chemoattractants, CXCL10 and CCL2, and neutrophil chemoattractants, CXCL8, CXCL1, and CXCL2, are consistent with these immune cells' infiltration into the lungs in SARS-CoV-2 infection leads to tissue damage (91).

The immune responses to SARS-CoV-2 infection and the role of the cytokines, chemokines, immune cells, and other mediators will be characterized with the final goal to identify targeted therapeutic strategies. Many clinical trials in the context of COVID-19 treatments confirmed this point of view, such as IL-6 blockers, IL-1 receptor antagonists, TNF-α inhibitors, JAK inhibitors, and corticosteroids (92–97).

In summary, abnormal and unregulated immune responses might be linked to the cytokine storm mechanism in COVID19, which is linked to disease severity. As a result, early cytokine storm management is critical for improving patient survival rates.

## JAK/STAT pathway in COVID-19

**Figure 2:**
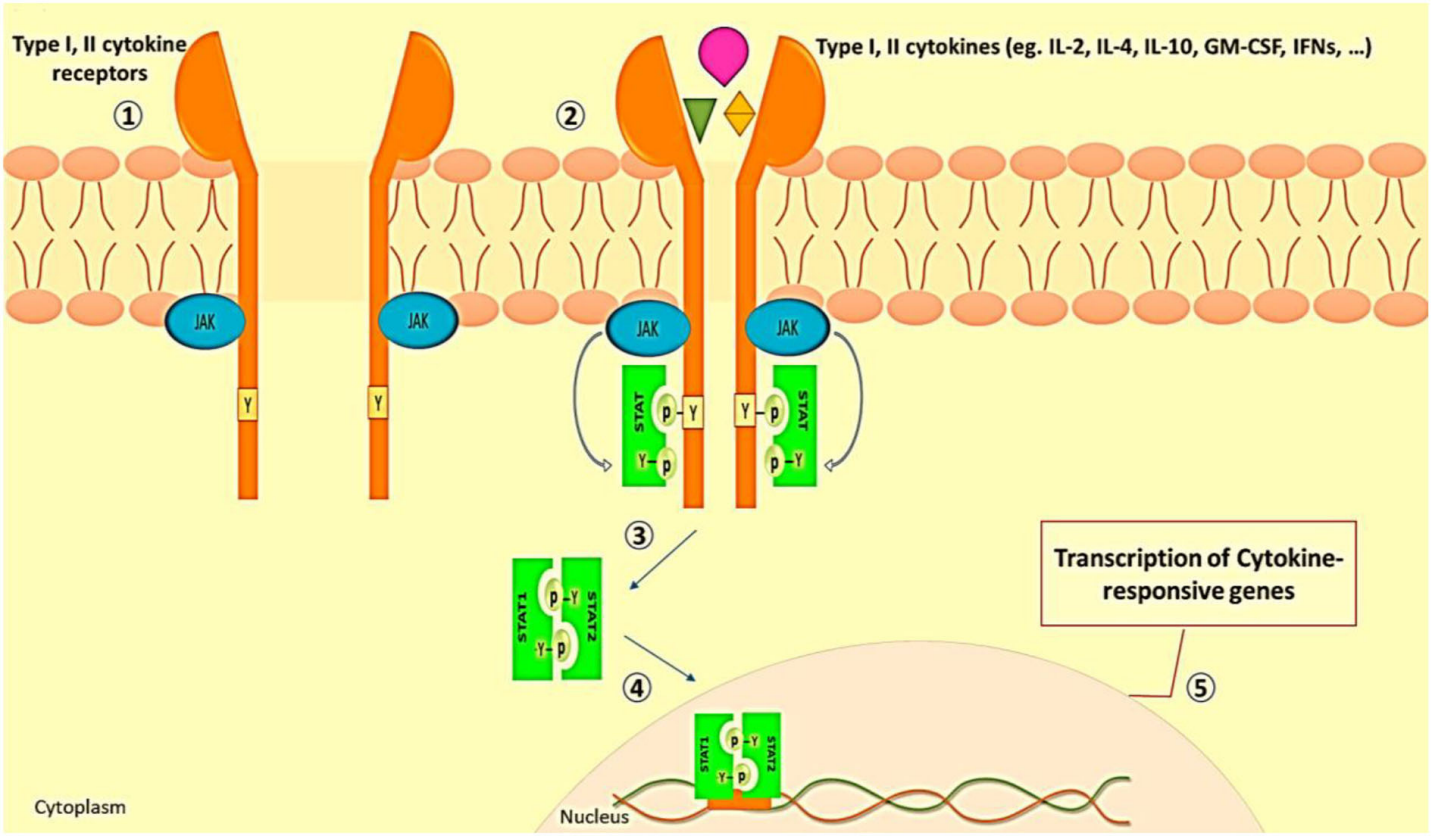
JAK/STAT signaling induction by type I and II cytokines. (1) In the resting state, the subunits of type I and II cytokine receptors are at a short distance from each other. (2) The engagement of ligand with receptors leads to receptor dimerization and JAK activation. (3) The activated JAKs phosphorylate the tyrosine residue of the receptor which recruits various members of STAT proteins. (4) Phosphorylated and activated STATs make themselves dimmer. (4) STATs complex translocates to the cell nucleus. (5) STATs bind to the promoter and lead to the transcription of cytokine-responsive genes (99).

As we discussed before, in severe instances of COVID-19, immunopathologic abnormalities such as downregulation of type I and type III interferon responses, as well as elevations in proinflammatory cytokine and chemokine production, are common (38). These events are reminiscent of the JAK/STAT pathway role in SARS-CoV-2 induced cytokine storm (98). The classic type of JAK/STAT pathway activation is related to cytokine receptor signaling. The induction of JAK/STAT signaling by type I and II cytokines is shown in Figure 2.

According to the researches, pneumocytes, pulmonary endothelial cells, and other immune cells are recruited *via* the JAK/STAT pathway into the inflammation sites during COVID-19 (100–103). For instance, STAT3 is involved in the differentiation and survival of CD4+ and CD8+ T cells. Also, facilitating the stimulation of CD8+ T cells in viral infection is mediated by STAT3 (104). Followed by cytokine production in NK cells, activated JAK1 downstream of IL-15 signaling regulates the development and growth of these cells. In the same way, the engagement of IL-2 and its receptor on NK cells, JAK1, 3 and STAT1, 3, 5 leads to cytotoxicity enhancement (105). Surprisingly, when IFN-α2 and IFN-γ (type I and III IFNs) are released, the expression of STAT1 increases, causing activation of the ACE2 receptors and, as a result, boosting SARS-CoV-2 entry into the host epithelium (101). Some interactions of SARS-CoV-2 with the JAK/STAT pathway are as follows:

In severe cases of COVID-19, the production of type I and III IFNs is insufficient in the early stages of the disease (38, 106). Studies revealed that patients with severe COVID-19 sustained type I IFN and pro-inflammatory responses in the late phase of the disease as compensatory mechanisms (86, 107). In confirmation with previous findings, Pairo-Castineira et al. revealed that the low expression of IFNAR2 (type I and III IFNs receptor subunit) and high expression of TYK2 (protein of Janus kinase family) are associated with life-threatening COVID-19 (108).

**Figure 3:**
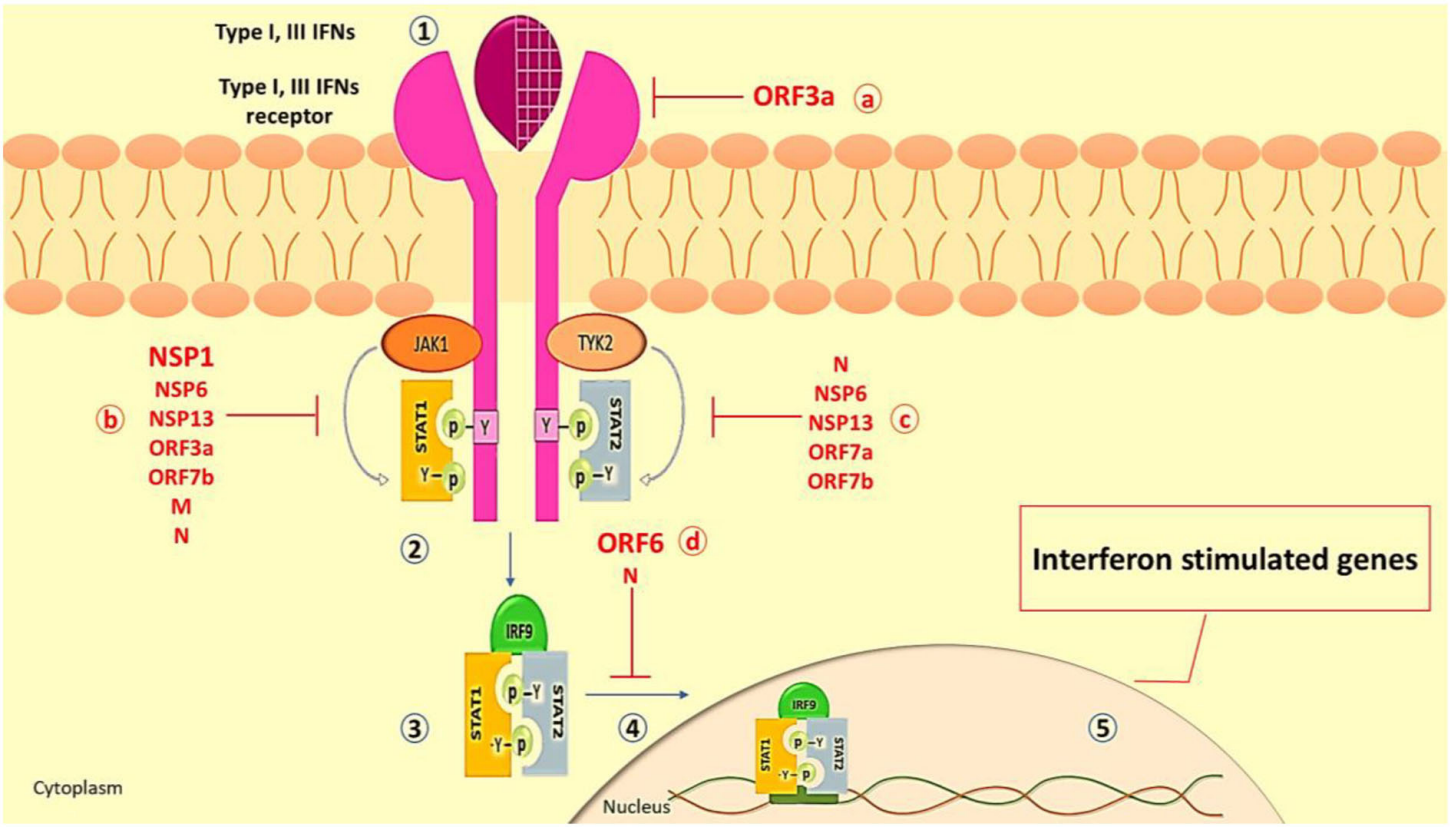
The Interferon type I and III canonical signaling pathway. (1) Type I and III IFNs (e.g., IFN-α, IFN-β, IFN-λ) released from virus-infected cells bind to their receptors (e.g., IFNA/LR) on neighboring cells, leading to the dimerization of receptors and activation of JAK1 and TYK2. (2) The activated JAK1 and TYK2 phosphorylate the tyrosine residue of the IFN receptor which recruits various members of the STAT family as STAT 1 and STAT2. Also, JAK1 and TYK2 add phosphorus to recruit STATs and activate them. (3) Following separation from the receptor, STAT1/STAT2 dimer interacts with IRF9. (4) The STAT1/STAT2/IRF9 complex moves to the nucleus. (5) Placement of the complex on its promoter on the gene leads to the robust expression of many classical IFN-stimulated genes (ISGs, such as ISG15, MxA, IFITM, etc.), which exert an antiviral role to restrict viral replication and spreading (109). SARS-CoV-2 inhibits IFN production and response through a variety of methods. As a result, target cells close to the original infection fail to receive essential and protective IFN signals, allowing the virus to propagate. The ORF3a can inhibit type III IFN receptors. The structural proteins of SARS-CoV-2, N, and M, and nonstructural proteins such as NSP1, NSP6, NSP13, ORF3a, and ORF7b quench IFN signaling by inhibition of STAT1 phosphorylation. (c) The phosphorylation of STAT2 in COVID-19 is repressed by N, NSP6, NSP13, ORF7a, and ORF7b proteins. (d) The translocation of the STAT1/STAT2/IRF9 into the nucleus is inhibited by N and ORF6 proteins which attenuate the transcription of the interferon-stimulated gene (13, 110, 111). IFN, Interferon; ISG, Interferon Stimulated Gene; STAT, Signal Transducer and activator of Transcription; IRF9, Interferon Regulatory Factor 9; TYK2, Tyrosine Kinase 2; JAK, Janus Kinase; MxA, Myxovirus Resistance Gene A; IFITM, Interferon Induced Transmembrane; OFR, Open Reading Frame; NSP, None Structural Protein.

During viral infection, canonical and non-canonical signaling pathways have been mentioned for IFN signaling. The IFN canonical signaling way, which is associated with JAK/STAT activation, is demonstrated in Figure 3. On the other hand, TLR-mediated or cytokines (mostly TNF) signaling pathways may redirect or synergize with IFN signaling, promoting the expression of non-canonical interferon-stimulated genes (non-ISGs) (112, 113).

Different proteins in SARS-CoV-1 interact with the actions of IFNs and JAK/STAT signaling (114). Based on genetic similarity between SARS-CoV-2 and SARS-CoV-1, SARS-CoV-2 may have the same antagonist activity (115, 116). Major SARS-CoV-2 proteins involved in IFN signaling disruption are ORF3a, ORF6, and NSP1 (103, 117) (Figure 3). Generally, SARS-CoV-2 inhibits STAT1 and IFNs, resulting in the activation of the STAT3 pathway as a dominant signaling pathway, which could result in COVID-19 complications. Regarding Yang et al.'s study, inhibition of STAT1 phosphorylation by SARS-CoV-2 results in a decrease in the transcription of the ISG in dendritic cells and macrophages (103). Once STAT1 function is impaired, it is possible that signaling via STAT3 pathways will compensate (118). STAT3 inhibits IFN response through several mechanisms, such as it binds to STAT1 and prevents the formation of STAT1/STAT1, inhibits binding of STAT1/STAT2/IRF9 to the DNA, and competes with STAT1 for binding to karyopherin alpha 1 (KPNA1, a nuclear translocation factor). Following viral infection, these circumstances would result in a greater functional STAT3:STAT1 ratio (119, 120). In hospitalized patients with COVID-19, some symptoms are related to aberrant STAT3 signaling such as hyperinflammatory condition, T cell lymphopenia, coagulopathy, and fibrotic status (90, 121, 122). In the context of hyperinflammation, STAT3 participates in the cytokine storm by enhancing cytokine production by co-activating the IL-6 amplifier (123).

## IL-6 signaling and intracellular pathway

IL-6 is a key mediator of intracellular defense against disease and injury, acting both as an indicator of multiple distinct types of cytokine storms and a host defense agent against infection (124, 125). Hyperinflammation, such as cytokine storms, can result from excessive IL-6 production (126). IL-6 is produced and secreted by different cells, such as macrophages and monocytes, and non-immune cells, including vascular endothelial cells, mesenchymal cells, and fibroblasts (127).

**Figure 4:**
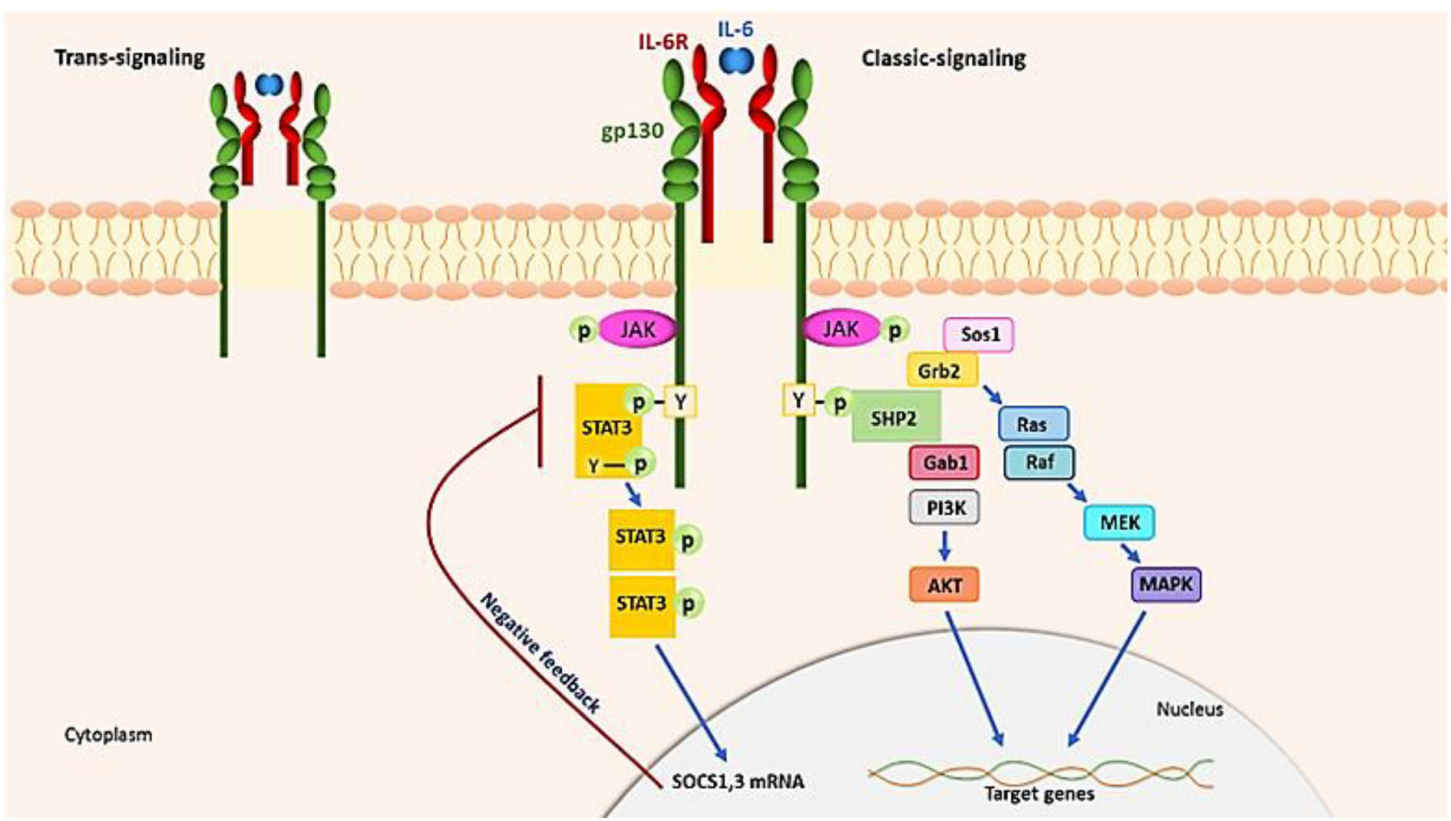
IL-6 signaling pathways. IL-6 requires two receptors, the IL-6R and the gp130, to begin signaling. IL-6 (classic signaling) binds to either fluid- or membrane-bound IL-6R (SIL-6R, trans-signaling). In two modalities of IL-6R signaling, a hexamer complex with gp130 is formed. The JAK–MAPK route and the JAK–STAT3 pathway are both used by IL-6 to transfer signaling. JAK kinases phosphorylate tyrosine in the cytoplasmic region of gp130, which promotes STAT3 phosphorylation and causes homodimerization, which works as a transcription factor in the nucleus.

The IL−6–SIL-6R complex also activates endothelial cells directly, releasing IL-6, IL-8, and MCP-1. This suggests that during a cytokine storm, IL-6 trans-signaling in the endothelium modifies the proinflammatory cytokine pathway. Therefore, IL-6 receptor-binding antibodies can be used to suppress hyperreactive immune responses (73, 128). To initiate signaling, IL-6 binds to either membrane-bound or circulating IL-6R (SIL-6R) in addition to a second glycoprotein, gp130 (59). IL-6 has pleiotropic properties related to its ubiquitous expression of gp130, whereas IL-6R is expressed solely by lymphocytes, monocytes and macrophages, and hepatocytes. Through two different signaling pathways, IL-6 promotes its effects by classic signaling and trans-signaling (129) (Figure 4). In two forms of IL-6R signaling, a hexamer complex with gp130 is produced. The JAK–STAT3 and the JAK–MAPK pathway are both used by IL-6 to send signals. The intracellular tyrosine motif in the gp130 intracellular region is responsible for biological activity. IL-6 signaling induces the production of two inhibitors, SOCS3 and SOCS1, which inhibit gp130 signaling activity (negative feedback). IL-6 attaches to a complex of mIL-6R and gp130 in traditional signaling and then activates the JAK-STAT3 pathway to relay signals. During trans-signaling, IL-6 binds to SIL-6R in the serum and tissue fluid. JAK kinases phosphorylate tyrosines in the cytoplasmic region of gp130 in response to IL-6. When JAK, which is normally attached to gp130, is phosphorylated by the stimulation of IL-6, it promotes STAT3 phosphorylation and homodimerization, allowing it to operate as a transcription factor in the nucleus, inducing the transcription of IL-6-responsive genes. JAK kinase also initiates the MAP kinase pathway by tyrosine phosphorylation of gp130 by binding SHP2. Different transcriptional actions are promoted by the JAK SHP2 MAPK pathway (130). According to that mentioned above, to reduce hyperinflammation conditions, the inhibition of the JAK/STAT and IL-6 signaling might seem to be a capable approach, but it should be used with caution in immunocompromised patients.

## JAK inhibitors as therapeutic agents against COVID-19

### JAK and IL-6 inhibitors

Due to the non-selective reduction of various cytokines' action (e.g., IL-2, IL-6, IL-7, and G-CSF), JAK inhibition is hypothesized to be able to decrease cytokine storm. Non-selective immune response inhibition, on the other hand, increases the likelihood of secondary infection. Since the JAK pathway influences STAT proteins, inhibiting the JAK system may result in STAT3-dependent cytokine storm inhibition (131). Many inflammatory illnesses, such as inflammatory bowel disease (IBD), rheumatoid arthritis (RA), and psoriasis, are treated with JAK inhibitors (132). Today, various antibodies that prevent IL-6, IL-6R, or associated signaling factors are used for a wide range of inflammatory diseases. Tocilizumab, a monoclonal antibody that directly targets IL-6R, has shown to be advantageous in treating cytokine storms caused by a variety of illnesses, Castleman's illness, CAR T-cell-induced cytokine storm, and COVID-19 (133–136). Critically, patients with COVID-19 experienced pneumonia-like symptoms and substantially elevated blood IL-6 levels. At the commencement of the pandemic, Wuhan physicians used off-label tocilizumab to manage COVID-19-infected hospitalized patients (137). A retrospective cohort study by Guaraldi et al. showed that in patients with severe COVID-19 pneumonia, therapy with tocilizumab, whether given intravenously or subcutaneously, may minimize the need for invasive mechanical ventilation or mortality (138). Rosas J. et al. indicated that therapy with baricitinib and tocilizumab in patients with COVID-19 hospitalized for interstitial pneumonia did not produce significant adverse effects and might be used to treat COVID-19-related interstitial pneumonia. (139). Also, Salama et al. showed tocilizumab decreased the probability of development to the composite endpoint of mechanical ventilation or death in hospitalized patients with COVID-19 pneumonia who no longer received mechanical ventilators; however, it did now not decorate survival (NCT04372186) (140). Stone et al. determined that tocilizumab became no longer powerful for stopping intubation or dying in moderately ill hospitalized patients with COVID-19 (NCT04356937) (141). Siltuximab and Sarilumab, both IL-6 antagonists, block both traditional and trans-signaling of IL-6. Furthermore, sgp130 could bind to IL−6–SIL−6R complexes and block the trans-signalingroute (142, 143). Table 1 summarizes IL-6/JAK/STAT3 inhibitors in COVID-19 (144, 145).

**Table 1:** Summary of IL-6/JAK/STAT3 inhibitors in COVID-19.

**JAK inhibitor**	**Function**
Baricitinib	JAK 1/2 inhibitor that has been shown to reduce inflammation and a treatment candidate for COVID-19 due to its potential anti-inflammatory and anti-viral effects.
Pacritinib	Oral activator (JAK2) and reduce the progression of acute respiratory distress syndrome and mechanical ventilation in hospitalized patients with COVID-19.
Ruxolitinib	Oral inhibitor of JAK and stops the IL-6 / JAK / STAT3 pathway that can significantly reduce IL-6 levels like upadacitinib and it can be a good treatment option for COVID-19.
Tofacitinib	Inhibits JAK3, JAK1, JAK2, and to a lesser extent TYK2 and inhibits receptor signaling through pairs of JAK2 and because it can suppress the production of different cytokines, such as IL-2, IL-7 and IL-6 can used for patients with COVID-19.
Nezulcitinib	Lung-selective inhibitor of the Janus kinases (JAKs), with potential anti-inflammatory and immunomodulatory activities, is a treatment for acute lung injury associated with COVID-19.
Upadacitinib	Targeting the JAK1 enzyme and decreases the activity of the immune system, reduce the expression of T-helper 2 and 22 cytokines and also the levels of interleukin6 (IL-6) like ruxolitinib that inhibits damage lung in patients with COVID-19.

### Baricitinib

Baricitinib is a JAK 1/2 inhibitor that has been shown to reduce inflammation in people with autoimmune disorders (146). Human anesthesia-related kinases are biochemically inhibited by baricitinib. The SARS-CoV-2 virus is caused by AAK1, Bike, and GAk genes (147). Baricitinib has also been shown to reduce multiple cytokines, which is suggested as a treatment for COVID-19 due to its potential anti-inflammatory and anti-viral effects (148). Baricitinib was approved by the Food and Drug Administration (FDA) in 2018 (149). It was used as an anti-rheumatic drug to treat adult patients with RA (150). It has also been demonstrated to help people with moderate to severe atopic dermatitis and reduce inflammation and itching (151). It was also used to treat a variety of autoimmune illnesses, including systemic lupus erythematosus (SLE) and atopic dermatitis (152). JAK is a tyrosine–protein kinase family and an intracellular enzyme that regulates immune cell function by modulating signals from cytokines and growth factor receptors (153). JAK proteins are classified into four groups (JAK 1, JAK 2, JAK 3, and TYK2). Various cell receptors link these proteins in different ways, resulting in heterodimers or homodimers (154). These JAK dimers phosphorylate transcription factors (STATs) and trigger the intracellular activities of inflammatory mediators, such as gene transcription, culminating in an autoimmune response (154). JAK2 and JAK1 have a greater affinity for baricitinib. It inhibits JAK proteins, prevents STATs from being phosphorylated and activated, and impacts the interferon, interleukin, and growth factor signaling pathways. Baricitinib suppresses JAK1/JAK2 expression and induces cell death in mutant cells (155).

Baricitinib plus remdesivir was better than remdesivir alone and had fewer serious side effects. It also aided in reducing recovery time and accelerating recovery, especially in patients receiving high-flow oxygen or non-invasive mechanical ventilation (156). Despite the lack of a significant decrease in disease progression, treatment with baricitinib in combination with standard therapy (including dexamethasone) may be useful. Several observational studies were performed, including small groups of hospitalized patients with COVID-19 and the elderly treated with baricitinib, and showed the first clinical evidence of improvement (133, 134). In another trial, baricitinib combined with remdesivir was found to be better than remdesivir alone in hospitalized people with COVID-19. Remdesivir produced a lot of negative side effects. The FDA granted baricitinib an emergency approval for patients with COVID-19 who needed oxygen support in the hospital (157). In the Marconi et al. study, patients received corticosteroids in the initial trial, which is now the standard of care for hospitalized patients in need of oxygen. Approximately 79 patients received systemic corticosteroids. About 19% of them received remdesivir. Eventually, 28 baricitinib and 30 (placebo) deaths occurred. However, the mortality was 28 days with baricitinib and 13 days with placebo. The benefits of baricitinib were more pronounced in patients who initially needed high-flow oxygen or ventilation. The incidence of serious side effects, including venous thromboembolism and serious infections, was similar in both groups (158). A study by Saber-Ayad et al. suggested baricitinib as a potential drug, which has a dual-mechanism drug, prevents the entry of SARS-CoV-2, and fights the cytokine storm that is the leading cause of death in COVID-19. However, subsequent studies of COVID-19 indicated that baricitinib treatment was only combined with ramedsivir. The study also showed that baricitinib-treated samples enriched baricitinib-suppressed transcripts in genes involved in the regulation of chemotaxis and the movement of immunological agents, such as neutrophils, eosinophils, monocytes, macrophages, granulocytes, natural killer cells, and lymphocytes, and also in the regulation of inflammation and humoral immune responses to viral and bacterial infections. CCL4 and family members of tumor necrosis factor (TNF, TNFSF11) were enriched. Baricitinib treatment appears to suppress tissue regeneration, ion, and membrane transduction, as well as cellular responses (159). A study by Hasan et al. showed that baricitinib could potentially suppress inflammatory cascades in severe COVID-19 pneumonia. A total of 238 patients with severe COVID-19 pneumonia were included in this prospective cohort research. For 14 days, 116 patients in the usual dose group and 122 patients in the high dose group were given 8–4 mg of baricitinib orally, and the clinical findings were compared between the groups. Baricitinib has been suggested as a viable anti-inflammatory medication for COVID-19 infection. The 8-mg daily dosage of baricitinib for 14 days led to early breathing normalization, lowered ICU and intubation needs, less 30-day mortality, and a lower 60-day readmission rate. According to this trial, fasting was superior to the standard 4-mg daily oral dosage of baricitinib in individuals with COVID-19 pneumonia (160). Baricitinib has been shown to improve the immune response to COVID-19 in several investigations. Baricitinib suppresses COVID-19-associated cytokine signaling *in vitro*, according to a study published by Stebbing et al. in 2020. They examined how GAK, AAK1, and BIKE were affected by a human numb-associated kinase (hNAK). Human viral infection is reduced (148). SARS-CoV-2 was mild to moderately suppressed by baricitinib and had a specific response with a substantial decrease in the number of cytokines, including IL-13, IL-1β, FGF, IL-10, IL-6, IP-10, IL-17, GM-CSF, IFN-γ, TNF-α, IL-4, IL-1ra, and MCP-1. Furthermore, baricitinib prevents viral endocytosis by inhibiting kinase signaling and inhibits cytokine release by blocking JAK1/2 signals. Baricitinib was rapidly transferred from intra-silicon studies to clinical trials (161). The study showed that healthy individuals treated with baricitinib had varying degrees of inhibition of cytokine-dependent phosphorylated STAT (pSTAT). Baricitinib inhibits signaling and also inhibits cytokines involved in COVID-19 infection, including IL-2, IL-6, IL10, IFN-c, and G-CSF. Baricitinib-induced cytokine-induced JAK/ TAT signaling resulted in a significant reduction in plasma IL-6 levels in patients with active RA who had a poor response to particular medications at week 12 (98). Baricitinib affects family members NAK AAK1, BIKE, and GAK STK16, and some facilitate the spread of coronavirus in the epithelial cells. Baricitinib showed a reduction in viral infection in infection of SARS-CoV-2 infected human liver treated with baricitinib (134). Four patients with bilateral COVID-19 pneumonia participated in this pilot study with varying degrees of illness severity; three of them were clinically unstable with moderate-to-severe illness. In March of 2020, they were all admitted from the emergency room to the ward. As would be predicted in patients with COVID-19 pneumonia, all patients showed detectable plasma IL-6 levels, as well as significantly elevated inflammatory markers (C-reactive protein [CRP]). In hospitalized patients, baricitinib therapy was associated with clinical and radiologic recovery, as well as a rapid reduction in SARS-CoV-2 viral load, inflammatory markers, and IL-6 levels. All patients had a significant detectable increase in plasma IL-6 levels (148). These findings suggest that baricitinib could be evaluated as a viable management option for patients with COVID-19 who are hospitalized and experiencing a cytokine storm and viral propagation. The discovery supports baricitinib's anti-cytokine profile and implies that it is a powerful AAK1/BIKE/GAK inhibitor that may diminish host cell infectivity. Baricitinib may reduce host cell virulence by inhibiting AAK1/BIKE/GAK in addition to confirming its anti-cytokine profile. Researchers found that baricitinib is a potent inhibitor of AAK1/BIKE/GAK and it affects host cell infectivity, confirming its anti-cytokine profile (148, 162). These data suggest that treatment with barsitinib was well tolerated in these four patients with COVID-19 bilateral pneumonia. There is evidence that treatment with baricitinib may reduce the inflammatory burden and may lead to a reduction in the severity of the disease in patients with COVID-19. This report showed that baricitinib is a cytokine inhibitor in patients with COVID-19 restricted to the intensive care unit. Baricitinib has previously been shown to inhibit IL-6 induction (148).

Only a few animal studies have been conducted on COVID-19, and one of those studies is Hoang et al.'s research from 2020. An 8-day treatment with baricitinib reduced macrophage production of cytokines and chemokines in the lung in a nonhuman primate animal model with SARS-CoV-2 (142). Goletti et al. (146) randomly assigned 1,033 patients to receive baricitinib and remdesivir (combined group, 515 participants) or placebo in their trial (control group, 518 patients). Remdesivir was given intravenously on day 1 at a loading dose of 200 mg, then 100 mg every day until discharge or death on day 10. For up to 14 days, baricitinib was given orally or via a nasogastric tube at a dose of 4 mg daily or 2 mg daily if renal function declined. Overall, the recovery time in the combined group was significantly shorter than the control group (mean, 7 days vs. 8 days; recovery rate ratio, 1.16; 95% confidence interval of the combined group as well as a 30% higher chance of improving the clinical condition per day). The combination group had a lower rate of significant adverse events than the control group. The combined group's recovery time was 10 days, while the control group's recovery time was 18 days. In addition, patients receiving combination therapy had the highest chance of improving their clinical condition. The highest documented efficacy of baricitinib was in patients with COVID-19 pneumonia who were receiving oxygen therapy but not invasive mechanical ventilation (146). The results of the Bronte et al. study showed that baricitinib affects the inhibition of cytokine-dependent pSTAT to varying degrees. Baricitinib inhibited cytokines involved in COVID-19 infection, including G-CSF, IFN-c, IL-6, IL10, and IL-2, (143). The study by Li et al. tested the antiviral activity of baricitinib by limiting the dose of baricitinib with a placebo. Twenty patients received baricitinib according to the study protocol. The control group was also considered. Patients were given 4 mg baricitinib twice daily for 2 days, then they received a low dose of 2 mg twice daily for 2 days, and finally 2 mg daily for patients over 75 years old, according to the inclusion criteria and pharmacokinetics of baricitinib. In patients with renal impairment, dose reduction was also investigated. The hepatotoxicity or myelotoxicity of patients in the baricitinib group was similar to that in the control group. Only one in 20 patients treated with baricitinib died after completing treatment, compared with 25 patients who died (45%) of the 56 patients in the group without baricitinib. Interestingly, patients treated with baricitinib had lower oxygen requirements compared to those from the control group (163). In the study by Pedro Abizanda et al., it was shown that patients from COVID-AGE groups who were hospitalized due to moderate to severe pneumonia were analyzed. Baricitinib treatment resulted in a significant reduction in mortality of up to 48% in patients 70 years of age or older, and an 18.5% reduction in the risk of 30-day absolute death (133). Patients who got a combination of bendamustine, lenalidomide, and dexamethasone (BRD) were enrolled in the Izumo trial. Baricitinib (14 days), dexamethasone (10 days), and remdesivir (10 days) were given to all patients. The effectiveness and side effects were assessed. In this investigation, 44 individuals with severe COVID-19 were enrolled. The 28-day mortality rate was only 2.3% (44.1 patients). For patients receiving BRD treatment, the mean hospital stay was 11 days, the recovery time was 9 days, length of stay in intensive care unit was 6 days, and duration of ventilation was they had 5 days of invasive mechanics and 5 days of supplemental oxygen therapy. Side effects occurred in 15 patients (34%). Iliopsoas hematoma, infectious endocarditis, liver dysfunction, herpes zoster, ventilator-associated pneumonia, thrombosis, and renal dysfunction, occurred in 2, 2, 11, 2, 2, 2, and 11% of patients, respectively. In severe instances of COVID-19, BRD was shown to be beneficial and had few adverse effects. The study's findings are positive. More randomized clinical trials, however, are required (164).

The aim of the study conducted by Perez-Alba et al. was to analyze the clinical outcome of dexamethasone or baricitinib and dexamethasone monotherapy in patients with severe COVID-19 pneumonia. A total of 596 patients were assessed for exclusion, with 793 being inclusion criteria. For the initial result, 197 patients were studied: 123 in the baricitinib plus dexamethasone group and 74 in the dexamethasone monotherapy group. Mechanical ventilation was used by 25.8% (197.51) and 42.9% (197.85) of patients needed ICU hospitalization. Overall, 27.9% (55/197) of people died after 30 days. The baricitinib plus dexamethasone group had a considerably reduced mortality rate than the dexamethasone monotherapy group. The two groups had the same number of nosocomial infections. Thirty-day mortality in COVID-19 pneumonia patients treated with baricitinib and dexamethasone was considerably lower than dexamethasone monotherapy. No differences in progression to invasive mechanical ventilation and nosocomial infections were observed. In hospitalized patients over 70 years of age with COVID-19 pneumonia, baricitinib is linked to an 18.5% lower risk of absolute death (130). Interestingly, a study of individuals treated with baricitinib found that ACE2 and TMPRSS2 levels were significantly reduced. In addition, baricitinib inhibits the AAK2 virus by binding to anesthetics-related kinases, and GAK-induced endocytosis, ultimately reducing the viral load (159). The ability of baricitinib to reduce the production of these cytokines, and hence immunological factors, has been demonstrated to aid patients with COVID-19 dramatically. For example, lowering serum cytokine levels, improving lymphocytes, and reducing the need for it has an oxygen flow behind it (165).

### Pacritinib

Pacritinib is an available oral activator (JAK2) and a mutant JAK2 JAK2V617F with potential anti-neoplastic activity (166). It competes for ATP binding to JAK2, which may lead to JAK2 activation, possibly the JAK-STAT output pathway, and thus become caspase-dependent apoptosis (135, 136).

According to CTI BioPharma, pacritinib's efficacy to reduce the progression of acute respiratory distress syndrome and mechanical ventilation in hospitalized patients with COVID-19 with severe coronavirus disease is being investigated in phase III clinical research. The cytokine storm, an inflammatory response that leads white blood cells to not only fight viral infections but also harm tissues, puts patients with severe COVID-19, especially those with cancer, at a significant risk for serious consequences of the condition. Pacritinib has the potential to avoid an inflammatory response to coronavirus infection and subsequent pulmonary insufficiency in the lungs, minimizing the requirement for a ventilator. Pacritinib is a kinase inhibitor for SCR1R, JAK2, and IRAK1 that is taken orally. The JAK family is required for normal blood cell growth and development, as well as the production of inflammatory cytokines and immunological responses. Blood malignancies, such as lymphoma, myeloproliferative neoplasms, and leukemia, appear to be linked to mutations in these kinases. Inflammatory and immune-mediated illnesses, such as acute graft-versus-host disease, may benefit from pacritinib treatment. Patients with COVID-19 have also been included in clinical trials to examine various cancer medications with potential therapeutic benefits for this patient population, including leronlimab (phase IIb/III), calquence (CALAVI study), and low-dose selinexor.

Researchers appear to be still exploring this medication according to various searches [198]. Pacritinib showed a satisfactory safety profile and pharmacological activity in preclinical investigations in hematologic malignancies and various cancers. Pacritinib significantly reduced the vitality and sphere-forming capacity of brain tumor-initiating cells (BTICs) *in vitro*, according to Jensen et al. Pacritinib was also observed to boost temozolomide activity in BTIC cells with unmethylated MGMT (O-6-methylguanine-DNA methyltransferase) promoters. Pacritinib has also been shown to be effective in treating glioblastoma multiforme (the JAK2/STAT3 inhibitor pacritinib effectively) (167). In addition, Brian and colleagues have shown that pacritinib selectively inhibits JAK2, both non-alloreactive T cells specific for nominal antigens and the development of beneficial Tregs, while limiting NK cell function in patients with COVID-19 (168). The most common side effects associated with pacritinib were thrombocytopenia and diarrhea, according to a large, randomized clinical trial (136). As of 2020, neither JAK2 inhibitor has resulted in severe infectious complications (136, 169). Furthermore, pacritinib suppresses Th1 cells that initiate cytokine release syndrome (CRS) pathogenesis through GM-CSF (pathogenic T cells and inflammatory monocytes incite inflammatory storm in severe patients with COVID-19).

Pacritinib's effects on AAK1 are unclear. Considering that fedratinib/pacitinib spare antigen-specific T-cell activity and minimize opportunistic infections, selective JAK2 inhibitors could be initially trialed rather than broader JAK1/2 inhibitors in the treatment of COVID-19 CRS (170).

In a recent study, Kabir et al. after screening and repurposing ~300 drugs, proposed twenty-seven candidates, including pacritinib, as probable candidates for further *in vitro* and *in vivo* research for the treatment of the SARS-CoV-2 infection.

Molecular docking was used to find compounds that will inhibit the identified molecular targets, TMPRSS2-ACE2 and SARS-CoV-2, from their own comprehensive database of roughly 300 highly described existing medications with proven safety profiles (203). Inhibitors that target SARS-CoV-2 proteases, the 3CLPRO and PLPRO, which are responsible for SARS-CoV-2 duplication and spread, are evaluated in the study by Jade et al. FDA-approved medications in the Zinc database, COVID-19 chemicals in the Maybridge database and Pubchem database against 3CLPRO and PLPRO proteases, and natural compounds in the Natural Product Activity database were used in silico high-throughput screening,. Furthermore, the study results show that pacritinib, bemcentinib, ergotamine, MFCD02180753, MFCD00832476, as well as the six chemicals that fight 3CLPRO bind with strong binding free energies to their active sites. As a result, these anti-PLPRO and anti-3CLPRO drugs could be employed to treat COVID-19 infections (171).

### Ruxolitinib

Ruxolitinib is an EU-approved bioavailable oral inhibitor of JAK for the treatment of myelofibrosis. Some research suggests that ruxolitinib lowers gene expression. Signal transducers and activators of transcription (STAT) linked to cytokine receptors regulate the inflammatory response (66).

It disrupts the ability of blood cells to form. JAK 2 triggers STAT activation and nuclear translocation by terminating kinase activity. Ruxolitinib stops the IL-6/JAK/STAT3 pathway and can significantly reduce IL-6 levels. Excessive inflammation prevents the reduction of lung failure in patients with COVID-19.

Furthermore, in patients with COVID-19 who are experiencing a cytokine storm as a result of the host reaction, this could be advantageous (144). Furthermore, ruxolitinib's anti-inflammatory and pro-apoptotic effects on senescent cells, which are hypothesized to be employed by COVID-19 to escape and change the immune system, could play a major role in COVID-19 treatment in patients with a poor prognosis, such as elderly people (145).

Ruxolitinib has applications in the treatment of severe COVID-19 disease and its complications such as respiratory distress syndroARDS. A phase II clinical trial study using ruxolitinib prevents multiple organ failure. Various therapeutic strategies have been adopted to modulate cytokine storms, such as IL-1 and IL-6 inhibitors. It is a JAK1 and JAK2 subtype inhibitor that is both powerful and selective.

A seventy-eight-year-old woman was diagnosed with COVID-19 pneumonia by PCR on oropharyngeal and nasopharyngeal swabs, and standard treatment with ruxolitinib 5 mg daily for 2 weeks was started. In the first 3 days of taking ruxolitinib, a slight improvement in lung function was observed. Ten days after starting treatment with ruxolitinib, she showed signs of paraesophageal and Centro lobular emphysema with a decrease in bilateral vitreous opacity and symptoms associated with pulmonary fibrosis. The patient tolerated ruxolitinib treatment well, as shown by the absence of thrombocytopenia, anemia, neutropenia, and opportunistic infections, which are the main side effects. These findings show that ruxolitinib not only improves lung function reduced by SARS-CoV-2 infection, but also reduces pulmonary fibrosis, improves tissue thickness and stiffness, and makes lung function less difficult (172).

The study by Giudice et al. indicated that 17 patients with ARDS related to SARS-CoV-2 were reported. The new combination of ruxolitinib 10 mg twice a day for 14 days and Eculizumab was used to treat these individuals. This combination resulted in a considerable reduction in D-dimer levels and a significant improvement in respiratory symptoms and radiographic lung abnormalities. The results support the combined use of ruxolitinib and Eculizumab for the treatment of ARDS (173). Furthermore, in the study by Innes et al., ruxolitinib showed promise as an anti-IL6 treatment for patients with COVID-19 infection with the severe syndrome. It was resistant to anti-IL6 therapy, but inhibiting JAK/STAT with ruxolitinib showed its safety and efficacy in these conditions (174). Anti-inflammatory drugs known as Jak inhibitors can help to reduce the overactive inflammatory reaction related to COVID-19.

The clinical data of 218 patients with COVID-19 who were hospitalized with severe pneumonia and treated with ruxolitinib were investigated.

The treatment period is given; results are given at 4, 7, 14, and 28 days. Retrospective data was gathered on clinical state, oxygen support demands, and laboratory markers. Overall, 66.5% percent of patients improved in follow-up, according to the physician's opinion. By day 7, 83.5% of them had made progress. Oxygen support status improved as well, with 21.6% of patients in ambient air on day 7 compared to 1.4% at baseline, rising to 48.2% on day 28. The reduction was significant. After 4 days an increase in CRP and lymphocyte count was observed, which appeared to be associated with a positive outcome. Approximately 87.2% of the patients were alive at the end of the observation period. There were no unexpected safety results, and patients reported minor side effects (175). In a study by Koschmieder et al. patients with COVID-19 were shown to be at risk for severe disease, especially those with comorbidities. Severe illness treatment infection with COVID-19 necessitates supportive intensive care. More specialized techniques are being considered, such as JAK inhibitors, which decrease the “cytokine storm.”

A 55-year-old patient with COVID-19 pneumonia used the JAK1/2 ruxolitinib inhibitor for primary myelofibrosis for 15 months before coronavirus infection. The patient had several serious underlying illnesses, including obesity, chronic renal disease, and arterial hypertension, and was at high risk for ARDS and COVID-19 mortality. The patient's condition remained unchanged without the requirement for mechanical ventilation because the abrupt removal of ruxolitinib could precipitate a lethal cytokine storm and ARDS. The patient tested negative for SARS-CoV-2 after 15 days and was discharged from the hospital. As a result, data suggest that ruxolitinib medication may be beneficial in preventing cytokine storms and ARDS in patients with COVID-19 pneumonia (176). COVID-19, which is caused by the novel SARS-CoV-2 virus, causes significant mortality and morbidity, and there is mounting evidence that inflammatory pathways play a role in lung injury. Severe patients with COVID-19 present an increase in inflammatory markers similar to secondary hemophagocytic lymphohistiocytosis (sHLH), which has been shown to predict mortality. There is evidence for the use of an interleukin inhibitor (IL-6) to suppress the inflammatory cytokine storm in this area. Ruxbeinib is effective in treating sHLH (147). The study by Saraceni et al. revealed that GVHD is one of the most common complications of lung damage caused by unregulated cytotoxic T cells and inflammatory cytokines. In another study, the selective JAK1/2 inhibitor ruxolitinib has shown promising results in this area. However, no clinical observations on the safety or efficacy of ruxolitinib therapy have been published in this field. In their study, Saraceni et al. described a case in which severe COVID-19 after hematopoietic stem cell transplantation in a patient with concurrent chronic GVHD, in which treatment with Ruxolitinib was well tolerated and positive (177). Ruxolitinib's immunomodulatory effect must be evaluated, and this medicine may raise the risk of opportunistic infections. In patients with an inflammatory state, disrupting the JAK signal transduction pathway could increase the risk of recognized adverse effects such as thrombocytopenia and anemia (178).

### Tofacitinib

Tofacitinib is a JAK kinase inhibitor that has no effect on other kinases in the human kinome. Tofacitinib inhibits JAK3, JAK1, JAK2, and to a lesser extent TYK2 in *in vitro* kinase assays. Tofacitinib preferentially inhibits pairwise JAK signaling in cellular settings through cytokine receptors associated with JAK1 and/or JAK3, but selectively suppresses receptor signaling through pairs of JAK2 (179).

Tofacitinib is a tiny chemical that controls cytokines that are critical in the course of inflammatory reactions and immunological responses in people with RA.

It suppresses the synthesis of cytokines that are needed for the advancement of immunological and inflammatory responses. Tofacitinib is a tiny molecule with a novel application in the treatment of RA. COVID-19 is presently being used in several clinical trials with tofacitinib. Yale University is conducting clinical research to determine the efficacy and safety of tofacitinib in patients with moderate COVID-19 (NCT04415151). Additionally, JAK inhibitors also reduce STAT1 activity, therefore their usage in patients with COVID-19 must be done with caution (98).

Upper respiratory tract infection, nasopharyngitis, headache, and diarrhea were adverse events (AEs) associated with tofacitinib while the most common serious AEs are described as serious infections such as herpes zoster, pneumonia, urinary tract infection, and cellulitis (151). Patients on tofacitinib have had gastrointestinal perforations, lymphoma, and malignancies (except non-melanoma skin cancer) (152). A total of 289 individuals with pneumonia were given tofacitinib 10 mg twice daily in the hospital for a maximum of 14 days or until discharge in a trial by Guimares et al. Death or respiratory failure by day 28 was the primary outcome. Glucocorticoids were given to 89.3% of the patients during their stay in the hospital through day 28, and the tofacitinib group had a cumulative incidence of 18.1% mortality or respiratory failure compared to 29.0 % in the placebo group. Serious side effects appeared in 20 (14.1%) individuals in the tofacitinib group and 17 (12.0%) patients in the placebo group. Patients with COVID-19 pneumonia had a decreased risk of respiratory failure or death for 28 days after receiving tofacitinib treatment. Adverse events were reported in 26.1 % of tofacitinib patients and 22.5 % of placebo patients. Ventricular tachycardia, acute myocardial infarction, deep vein thrombosis, myocarditis, and ventricular tachycardia were all reported by individuals in the tofacitinib group as side effects. Each patient in the placebo group suffered from heart failure and hemorrhagic stroke. Serious infection occurred in 4.2% of the placebo group and 3.5% in the tofacitinib group (153). According to the findings of the study by Agrawal et al., out of 2,326 patients, 37 (1.6%) were treated with tofacitinib. Patients who got <20 mg of tofacitinib per day were 17 (45.9%) and 20 (54.1%), respectively. Tofacitinib was administered to 17 (45.9%) and 20 (54.1%) patients, respectively, in doses of 20% and <20 mg total daily. Thirty tofacitinib patients (81.1%) had ulcerative colitis compared to 946 (41.3%) patients on other IBD medications. In comparison to other medications, tofacitinib patients recovered significantly less (154). Maslennikov et al. included 32 patients in their trial who received tofacitinib and 30 individuals who did not receive any anti-cytokine medicines (control group). The tofacitinib group had a lower death rate and a lower ICU intake rate compared to the control group. The tofacitinib group had a much-reduced volume of the affected lung and a significantly higher oxygen saturation than the control group up to 10 days following delivery. CRP levels in tofacitinib patients were lower than in control patients. In the COVID-19 study, tofacitinib was proven to be a safe and effective treatment for cytokine release syndrome [221]. Alopecia areata, an autoimmune disease, damages the epithelium of the hair follicle. As a result, hair loss occurs. JAK3 has been demonstrated to have a key role in the disease's etiology. Tofacitinib is a JAK3 and JAK1 inhibitor that can suppress the production of different cytokines, such as IL-2, IL-7, and IL-6. Oral tofacitinib has been found in numerous studies to help alopecia areata sufferers regrow their hair. As a result of the recent COVID-19 outbreak, avoidance of JAK inhibitors during active infection has been suggested to prevent possible immunosuppression.

Ferreira et al. indicated that two alopecia patients with active COVID-19 infection who continued to use tofacitinib saw no deterioration of their condition (155). Moiseev et al. conducted the study to see if tofacitinib can reduce the probability of individuals with COVID-19 needing invasive mechanical dilation or dying. Patients with COVID-19 with low oxygen saturation and a prolonged fever were enrolled. The trial included 384 patients with COVID-19, 253 of whom were treated with conventional tofacitinib alone. In the tofacitinib and control groups, 12.5% (72.9) and 14.1% (185.26) of patients who needed respiratory assistance commenced mechanical ventilation or died during hospitalization, respectively (180). Hayek et al. looked at the effects of tofacitinib in combination with dexamethasone in hospitalized COVID-19-associated pneumonia patients. Tofacitinib was given universally to 138 (51.3%) of the 269 eligible patients, whereas dexamethasone was given to 131 (48.7%). A total of 44 individuals died, with 30 (68.2%) dying on dexamethasone and 14 (31.8%) dying on tofacitinib.

Tofacitinib and dexamethasone both had mortality rates of 10.1 and 22.9%, respectively. After controlling for age and clinical factors collected in the hospital, the tofacitinib group had a 70% lower chance of mortality than the dexamethasone group. With the addition of dexamethasone, COVID-19 pneumonia treatment improved quickly. This study demonstrated the potential benefits of tofacitinib in COVID-19 pneumonia. In COVID-19 pneumonia, the inclusion of tofacitinib in a therapy regimen containing dexamethasone has the potential to improve survival when compared to dexamethasone alone (181).

### Nezulcitinib

Nezulcitinib is a lung-selective inhibitor of the Janus kinases (JAKs), with potential anti-inflammatory and immunomodulatory activities (182). It is a panJAKi with IC50s of 10.3 nM, 10.6 nM, 10.2 nM, and 9.2 nM for JAK1, JAK2, JAK3, and TYK2, respectively, that can be administered by inhalation, and it inhibits the activity of the JAKs, thereby disrupting cytokine-induced activation of JAK-STAT signaling pathways in the airways (157). Nezulcitinib, designed for inhalation, is for the development for the treatment of acute lung injury associated with COVID-19 (182).

### Upadacitinib

Upadacitinib is in a class of inhibitors medications called Janus kinase (JAK), targeting the JAK1 enzyme and decreasing the activity of the immune system (183). It is a group of four tyrosine kinases (JAK1, JAK2, JAK3, and TYK2) involved in the process of immune-mediated inflammatory diseases (IMIDs). In patients with COVID-19, IL-6 plays a role in the cytokine storm that damages the lungs. Disruption of JAK1 signaling induced by upatacitinib reduced not only the expression of T-helper 2 and 22 cytokines but also the levels of interleukin6 (IL-6), through inhibition of STAT3 phosphorylation (199).

## Possible adverse effects of drugs that affect JAK-STAT signaling during COVID-19

Baricitinib is generally regarded as safe and acceptable; however, because it is an immunosuppressive medicine, it can raise the risk of serious infections (184). Urinary and upper respiratory tract infections are the most common. But herpes zoster infections are becoming more common (185). Candidiasis, pneumocystosis, tuberculosis, histoplasmosis, CMV infections, and BK virus infection are among the opportunistic illnesses that have been recorded (184).

Upper respiratory infection, nasopharyngitis, and headache are some of the side effects (186). They are prevalent among baristinib patients. The use of baricitinib for an extended period of time may increase the risk of thromboembolic illness in patients. Bacterial infections, viral infections, pneumocystis, herpes zoster, urinary tract infections, acute histoplasmosis, fungal infections (candidiasis), cryptococcosis, and pneumonia, are among the other side effects (187). Baricitinib has been linked to bone marrow suppression as well as blood abnormalities including lymphopenia, neutropenia, and anemia (186).

An increase in medium cholesterol, low-density lipoproteins (LDL), and high-density lipoproteins (HDL), without an increase in the ratio of HDL to LDL, is another side effect that usually occurs after 12 weeks of using baricitinib (188). Some people had elevated CPK levels. In clinical trials, pulmonary embolism (PE) and deep vein thrombosis (DVT) were also detected (189). Malignancies such as skin cancer and lymphoma struck a tiny fraction of the participants. Stomach discomfort, nausea, and vomiting are some of the most prevalent gastrointestinal problems (190). Severe adverse effects including gastrointestinal perforation, on the other hand, are uncommon but have been recorded in patients who had previously had diverticulitis (191). The use of baricitinib in pregnancy has not yet been extensively studied (190). In animal research, baricitinib has been found to reduce fetal weight and teratogenicity. Baricitinib was detected in rat milk throughout tests. It should be avoided by breastfeeding mothers because there is no data on its presence in human milk. Baricitinib has also been shown in animal tests to harm female fertility while having no impact on male sperm generation. Ruxolitinib has the potential to cause liver damage, hematological issues, and ambiguity. Infection care is required based on reports of viral/bacterial reactivation in patients. Hepatotoxicity of grade 3 is experienced by a patient. Two patients with anemia already had grade 3 anemia, which was treated with periodic blood samples in the ICU (191). Diarrhea, bloating, a sense of constant movement in one's body or surroundings or full feelings of a spinning sensation, skin rash, weight gain (192), and other common side effects are all common.

## Conclusion

The cytokine storm in COVID-19 is caused by the interaction of multiple immunological components, including interleukins, chemokines, TNF-α, and IFNs. Various drugs have effective potentials in overcoming problems due to the hyperactivation of the immune system associated with the JAK/STAT signaling pathway.

This signaling pathway mediates cellular responses to proinflammatory cytokines. Therefore, inhibition of the JAK/STAT pathway may induce the inhibition of various cellular responses in COVID-19 infection.

In conclusion, JAK inhibitors including baricitinib, pacritinib, ruxolitinib, and tofacitinib have beneficial potential in the recovery and reduction of novel coronavirus disease due to anti-inflammatory and anti-viral effects.

## Author contributions

PO, NB, MKh, and FS: design of study. PO, NB, MKh, FS, SY, and MR: acquisition of data. PO, MKh, SY, MR, MM, HA, and ZE: evaluation of data and preparation of the manuscript. PO, NB, MKh, FS, MKo, and FA: assessment of data. All authors read and approved the final manuscript.

## Conflict of interest

The authors declare that the research was conducted in the absence of any commercial or financial relationships that could be construed as a potential conflict of interest.

## Publisher's note

All claims expressed in this article are solely those of the authors and do not necessarily represent those of their affiliated organizations, or those of the publisher, the editors and the reviewers. Any product that may be evaluated in this article, or claim that may be made by its manufacturer, is not guaranteed or endorsed by the publisher.
